# Transplacental Nutrient Transport Mechanisms of Intrauterine Growth Restriction in Rodent Models and Humans

**DOI:** 10.3389/fphys.2017.00951

**Published:** 2017-11-27

**Authors:** Elke Winterhager, Alexandra Gellhaus

**Affiliations:** ^1^Electron Microscopy Unit, Imaging Center Essen, University Hospital, University of Duisburg–Essen, Essen, Germany; ^2^Department of Gynecology and Obstetrics, University Hospital, University of Duisburg–Essen, Essen, Germany

**Keywords:** intrauterine growth restriction, placenta, glucose, amino acid, fatty acids, transport, human, rodent

## Abstract

Although the causes of intrauterine growth restriction (IUGR) have been intensively investigated, important information is still lacking about the role of the placenta as a link from adverse maternal environment to adverse pregnancy outcomes of IUGR and preterm birth. IUGR is associated with an increased risk of cardiovascular, metabolic, and neurological diseases later in life. Determination of the most important pathways that regulate transplacental transport systems is necessary for identifying marker genes as diagnostic tools and for developing drugs that target the molecular pathways. Besides oxygen, the main nutrients required for appropriate fetal development and growth are glucose, amino acids, and fatty acids. Dysfunction in transplacental transport is caused by impairments in both placental morphology and blood flow, as well as by factors such as alterations in the expression of insulin-like growth factors and changes in the mTOR signaling pathway leading to a change in nutrient transport. Animal models are important tools for systematically studying such complex events. Debate centers on whether the rodent placenta is an appropriate tool for investigating the alterations in the human placenta that result in IUGR. This review provides an overview of the alterations in expression and activity of nutrient transporters and alterations in signaling associated with IUGR and compares these findings in rodents and humans. In general, the data obtained by studies of the various types of rodent and human nutrient transporters are similar. However, direct comparison is complicated by the fact that the results of such studies are controversial even within the same species, making the interpretation of the results challenging. This difficulty could be due to the absence of guidelines of the experimental design and, especially in humans, the use of trophoblast cell culture studies instead of clinical trials. Nonetheless, developing new therapy concepts for IUGR will require the use of animal models for gathering robust data about mechanisms leading to IUGR and for testing the effectiveness and safety of the intervention among pregnant women.

## Introduction

Doerner (1973) as well as Barker and co-workers (Barker and Osmond, 1986; Hales and Barker, 1992) first developed the concept that an adverse maternal environment during the sensitive phase of intrauterine development is associated with a predisposition toward a host of various diseases in later life, a hypothesis later called the Developmental Origins of Health and Disease (DOHaD). Since then, scientists have been challenged to evaluate the molecular mechanisms that are initiated by an insult during intrauterine development and that lead to chronic diseases in adulthood.

The fundamental epidemiological observations about the programming of health and disease *in utero* were performed by Barker and co-workers using data from England and Wales. In Wales, infants born between 1921 and 1925, a period of starvation in that country, exhibited high rates of infant mortality and low birth weight. Additional evaluation of data obtained between 1968 and 1978, when these children had reached adulthood, demonstrated a two-fold higher risk of heart disease, chronic bronchitis, and emphysema among this group (Barker and Osmond, 1986). The findings of this epidemiological study were corroborated by those of another cohort study involving Dutch women whose food intake had been severely reduced during pregnancy because of terrible conditions during World War II (Smith, 1947). Studies involving their children were performed years later by Ravelli et al. (1976) and Roseboom et al. (2000). These studies detected a significant increase in the incidence of heart disease and adiposity. Taken together, these mechanisms are called *fetal programming*.

Fortunately, today's researchers rarely find such well-defined endemic cohorts of pregnant women living under similar social and economic conditions in industrial countries, experiencing undernutrition during the sensitive phase of fetal development, and allowing the possibility of a follow-up study. Instead, other insults, such as high body mass index, gestational weight gain, eating disorders, alterations of the immune system, as well as high blood pressure, infection/inflammation and hormonal imbalance, have been raised that may seriously affect the offspring, even over several generations. Furthermore, maternal life style factors such as stress, smoking, diet, and drugs contribute to fetal programming. Thus, it is difficult to determine the factors leading to intrauterine growth restriction (IUGR) and the consequences of this condition in terms of diseases among humans in later life. The diseases that occur later in life because of this reprogramming are manifold and comprise metabolic syndromes such as cardiovascular disease, diabetes, obesity, dyslipidemia (Barker, 1995; Eriksson et al., 2001; Zohdi et al., 2014), and chronic immune disease, as well as neurophysiological disorders (Nepomnyaschy and Reichman, 2006; Ananth and Friedman, 2014; Murray et al., 2015). Currently, it is impossible to predict which maternal insult could lead to a defined placental dysfunction resulting in a specific disease, or whether various insults affect similar molecular pathways and lead to fetal reprogramming and the lifelong burden of a group of diseases.

It must be considered that the adverse programming effect on fetus and offspring is not only restricted to this intrauterine period of development but also can occur at the time of fertilization, predominantly with *in vitro* fertilization (IVF) (Scherrer et al., 2015; Sartori et al., 2016), or postnatally because of lifestyle factors and needs to be discriminated from IUGR.

IUGR is defined as a significant reduction in the fetal growth rate resulting in a birth weight below the tenth percentile for gestational age (GA) and is estimated to occur in as many as 10% of all pregnancies (Alfirevic and Neilson, 1995; Resnik, 2002; Romo et al., 2009). It is essential to distinguish between infants who are constitutionally small for gestational age (SGA), mostly attributable to genetic background and those who have experienced IUGR, mostly caused by placental insufficiency (Lockwood and Weiner, 1986; Patterson and Pouliot, 1987). Children affected by IUGR experience reductions in quality of life and also place a large burden on health care systems and society. Approximately 5–10% of all IUGR pregnancies result in stillbirth or neonatal death. However, it is an oversimplification to consider low birth weight as a reflector of fetal programing *in utero*, because the mechanisms leading to IUGR, which is usually caused by placental insufficiency, are unknown and are probably multifactorial. IUGR is combined with inadequate growth of organs essential for later health and development, and infants affected by asymmetrical IUGR appear to be more prone to short-term and long-term disease than are infants with symmetrical IUGR (Patterson and Pouliot, 1987; McIntire et al., 1999; Devaskar and Chu, 2016).

Appropriate embryonic development requires a precise cell-lineage allocation that is highly regulated both spatially and temporally within a network of gene expression in combination with precise cell signaling pathways.

In addition to this temporally and spatially controlled lineage-specific gene expression pattern, DNA maturation by epigenetic modification is necessary for printing an epigenetic memory of the gene pool. Moreover, endogenous programs combined with the maternal environment govern the individual metabolic properties of cells, organs, and fetus. Adverse stimuli and noxious events during this sensitive phase of development interfere with this balance of rapid proliferation and differentiation of the architecture and function of fetal organs and metabolic programming.

## Role of the placenta in IUGR

During the intrauterine period, most insults do not directly affect the fetus but instead first alter placental development and function. As a consequence, altered fetal development, if not attributable to structural or genetic fetal defects, is associated with altered placental physiology. A growing body of evidence underscores the importance of placental dysfunction as a key link between adverse maternal factors and lifelong impairments to the health of the offspring. IUGR can be caused by reductions in placental blood flow and exchange area of the placenta, as well as changes in the placental transporter system. Permeable membrane substances are greatly influenced by these factors (Gude et al., 2004) because the concentration gradients and the size and permeability of nutrients depend not only on the volume of maternal blood flow or the plasma membrane area of the trophoblast but also on membrane-bound transporters across the placental barrier.

Under physiological conditions, the placenta regulates the delivery of nutrients and oxygen to the fetus and disposes of waste in adaptation to the maternal environment. However, placental plasticity seems to fail under extreme environmental situations, and the placenta itself becomes diseased. Even a less severe adverse environment, such as maternal undernutrition, diet, and overfeeding, can impair placental physiology (Wallace et al., 1996; Kavitha et al., 2014). Most IUGR pregnancies are attributable to placental disease that leads to disturbances *in utero*-placental blood flow, causing chronically hypoglycemic placentas (Redline, 2008). Other placental factors are responsible for malnutrition and ischemia; they include abnormal trophoblast invasion resulting in an insufficient remodeling of the spiral arteries, with reduced placental circulation in preeclampsia. Moreover, impairments in placental development or differentiation of the various trophoblast cell subpopulations, as well as reduced placental size or altered placental nutrient transport capability lead to alterations in immune cell population and hormone production, which in turn contribute to placental malfunction. Alterations in placental physiology such as those described above can be caused by epigenetic or genetic modifications (Tarrade et al., 2015). However, our knowledge about placental structure and function throughout pregnancy remains limited, and the molecular mechanisms leading to placental insufficiency and resulting in IUGR are largely unknown and no treatments for IUGR are currently available. Comprehensive and well-defined investigations and experiments are necessary for elucidating these complex alterations in placental function, such as changes in the transporter system upon well-defined maternal insults, and for obtaining stricter diagnostic criteria for the interplay between placenta and IUGR.

The causes and consequences of IUGR must be looked at in their entirety, beginning with the insults, the consequences for placental physiology, and the impact on the various fetal organs. Such studies require translational science approaches for examining reproductive and neonatal factors, as well as follow-up studies. However, this translational experimental approach is hampered by the fact that worldwide comprehensive biobanks with defined placental tissue from IUGR pregnancies are rare, and only small cohorts of children with IUGR are available for follow-up studies (Kvehaugen et al., 2011). Because of the urgency of this topic and growing evidence of human disease programming early *in utero*, the use of new technologies *in vivo* has become an important focus, resulting in the creation of the Human Placenta Project of the National Institute of Child Health and Human Development (https://www.nichd.nih.gov/research/HPP/Pages/default.aspx), which monitors placental growth and function for a better understanding of the entire course of pregnancy, especially the earlier gestational period (Guttmacher et al., 2014). However, adequate animal models are absolutely necessary for addressing complex questions about DOHaD; about the key events leading to alterations in placental function that cause IUGR; about the effects of IUGR on fetal organ impairment; and about medical monitoring and possible interventions during pregnancy. This review will address both, rodent models and the results of human studies that have investigated the molecular causes of IUGR, which mainly result from alterations in diet, reductions in the oxygen supply, or both, with a focus on placental transporter systems.

## Animal models of IUGR

Relevant animal models have been widely used to elucidate the mechanisms and molecular events that link an adverse maternal environment to alterations in placental physiology leading to IUGR offspring (Swanson and David, 2015).

**Figure 1:**
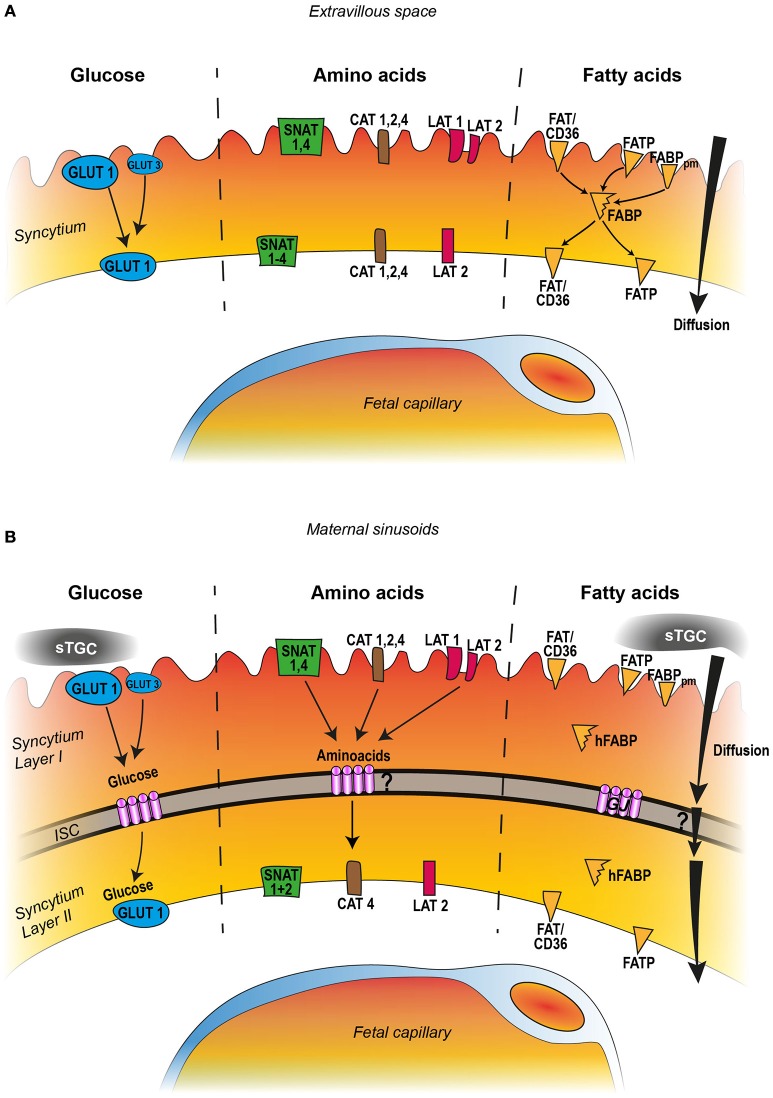
Comparison of human **(A)** and rodent **(B)** nutrient transport across the placental barrier, featuring the syncytial trophoblast layer. The transporter categories are manifold and only the most important and well documented transporters are indicated in the figures which include GLUT1, GLUT3, for glucose; SNAT1-4, CAT1, 2, 4, and LAT1,−2 for amino acid transport; for fatty acids FAT/CD36, FATP, and the fatty binding proteins FABPpm and FABP. The human placenta barrier consists of one syncytial trophoblast layer. The key molecules glucose, amino acids, and fatty acids are taken up from the maternal blood (extravillous space) by specific transporter systems and are released at the basal cell membrane into the fetal compartment. Glucose transport occurs toward the concentration gradient whereas amino acid transfer is an active process against the concentration gradient. Fatty acids may diffuse along the concentration gradient but in addition are taken up by a transporter system and translocated to the plasma membranes with help of the fatty acid binding protein (FABP). The rodent placental barrier of the labyrinth demonstrates three trophoblast layers: sinusoidal trophoblast giant cells (sTGS), which constitute no barrier function for nutrient transport, and two syncytiotrophoblast layers, layer I and layer II, separated by an intercellular space (ISC). The ISC is tightly bridged by numerous gap junction channels (GJ). Similar to the human situation glucose, amino acids, and fatty acids are taken up from the maternal sinusoids and translocated to the basal membrane of layer II with the help of a similar transporter system. Except from glucose which crosses the space through gap junction channels, it remains elusive how the other nutrients can cross the intercellular space.

It is under debate which animal model is most suitable for studying human placentation and the placental disorders associated with IUGR. Since at least the publication of a textbook by Grosser (1909), placentologists have been aware that both placentation and the anatomy and development of the placenta are highly species-specific. However, selecting the preferred animal model depends on the questions asked. Although no single animal model can encompass the multifactorial causes and consequences of IUGR, much recent knowledge has been derived from studies using rodents to mimic impaired maternal or placental factors (Grigsby, 2016). Rodents have the advantage of a short gestational period and a large litter size that facilitate generation studies leading to statistically secured data. Moreover, like humans, rodents exhibit an invasive type of implantation leading to a hemochorial placenta with a discoidal shape. The true placental barrier is formed only by trophoblast layers facing maternal blood spaces. However, in contrast to humans, in rodents the main cellular barrier is formed by two syncytial layers rather than by a single syncytiotrophoblast layer. Thus the transport of nutrients across the trophoblast is more intricate in rodents, with two syncytial compartments, than in humans. In rodents, nutrients must cross the sinusoidal trophoblast giant cells, the syncytiotrophoblast, and the endothelium of the fetal capillaries to reach embryonic blood vessels; in humans the syncytiotrophoblast is the only true membrane barrier (Figures 1A,B). In both humans and rodents, giant cells play a key role in decidua and vessel invasion at the maternal-fetal interface. Despite structural differences between the human and rodent placentas, they also have many overlapping functional characteristics that are important for deciphering placental nutrient transfer in humans. It is also advantageous that the maintenance of pregnancy is guaranteed by progesterone produced in the placental giant cells.

A further advantage of the rodent model is that genetically modified mice can be generated for studies elucidating the signaling cascades involved in poor pregnancy outcome, if these mice mimic one or more factors that can be used to unscramble specific or common pathways.

A large advantage of using mouse models is the availability of mouse trophoblast stem cells (TSCs) that are generated from normal or genetically manipulated mice blastocysts or ectoplacental cone cells (Tanaka et al., 1998); the generation of these cells from humans has not been successful to date. Those murine cell lines can be shifted into trophoblast lineage differentiation, and this shift results in the various subpopulations of placental tissue. TSCs are an ideal system for testing the influences of insults such as hypoxia (Koch et al., 2012; Xie et al., 2014) and dysfunction of signaling cascades. Meanwhile, several rodent models of IUGR exist, and these models can mimic phenotypes of IUGR in humans, such as alterations in placental morphology and nutrient transport (Dilworth and Sibley, 2013).

One of the most common rodent models used to mimic the influence on placental development and offspring is reduced or altered intake of food, such as a low-protein or high-fat diet, or vessel ligation during gestation. A consistent finding in rats (Woodall et al., 1996; McMullen and Langley-Evans, 2005) and mice (Kawamura et al., 2007) is that fetuses exposed to undernutrition or a low-protein diet during the first half of pregnancy or throughout pregnancy are significantly smaller than normal. Even a less severe low-protein diet that does not induce IUGR leads to similar alterations in kidney morphology, with a reduction in the number of glomeruli and glomerular necrosis (Yuasa et al., 2016). The other animal model used for IUGR is the artery ligation model, which mimics an acute situation and not only diminishes the nutrient supply but also leads to oxygen deprivation (Janot et al., 2014).

## Comparison of placental transport mechanisms of rodents and humans with IUGR

### Glucose transport across the placental barrier

The placenta acts as a nutrient sensor by adapting the transport system according to the maternal environment. To date, several reviews have described the expression of the isoforms GLUT1, GLUT3, and GLUT4 in placental tissue from both humans and mice and have suggested how the function of each isoform contributes to the metabolic needs of the placenta (Illsley, 2000; Lager and Powell, 2012).

Because glucose is the primary nutrient required for growth of the fetus and placenta, glucose is the most prominent factor responsible for IUGR. The fetus depends on the uptake of glucose from the maternal circulation (Devaskar and Chu, 2016) across the placenta (Lager and Powell, 2012) by the glucose transporters of the GLUT family. The primary placental glucose transporter GLUT1 (*Slc2a1*) is the predominant isoform abundantly expressed during early pregnancy and at term in humans (Sakata et al., 1995) and rodents (Das et al., 1998). The GLUT1 protein is highly expressed throughout pregnancy and is located more in the apical than in the basal membrane of the syncytiotrophoblast (Carruthers et al., 2009). In addition to GLUT1, GLUT3 (*Slc2a3)*, which has a high affinity for glucose (Jansson et al., 1993), is present in human placentas during the first and second trimesters, a finding suggesting that GLUT3 may play a role in glucose uptake early in pregnancy. GLUT3 is only detected in the microvillus membrane but not in the basal plasma membrane of the syncytiotrophoblast (Brown et al., 2011) (Figure 1A). As in humans, GLUT1 is abundantly expressed in all placental cell populations in rat placentas, predominantly in the syncytiotrophoblast facing the maternal sinusoids, as is GLUT3 to a lesser extent (Zhou and Bondy, 1993). Like in humans GLUT3 is exclusively found in the plasma membrane of the syncytiotrophoblast layer lining the maternal blood side (Shin et al., 1997, Figure 1B).

However, some differences in glucose transport between rat and human placentas must be mentioned. Rats and mice exhibit a prolonged period of nutrient supply via the yolk sac placenta during the first trimester of pregnancy. As a consequence, GLUT1 is found in epithelial cells of both visceral and parietal yolk sac vessels (Hahn et al., 1995). Moreover, because rodents have a two-layered syncytiotrophoblast, nutrients must cross both layers, which are separated by an intercellular space. But instead of using GLUT transporters in the basal membrane of layer I and in the apical membrane of layer II, the rodent placenta solves this problem by bridging the gap directly with numerous gap junction channels composed of connexin26. This intercellular channel serves as a diffusion channel for glucose, as evidenced by the connexin26 knock-out mouse model; these mice die *in utero* at around embryonic day 10 of pregnancy because of severely reduced glucose transfer into the embryo at a time when large amounts of glucose are needed for rapid fetal growth (Takata and Hirano, 1997; Gabriel et al., 1998, Figure 1B).

In mice, deletion of GLUT1 results in embryonic loss before ED12 (Ohtsuki et al., 2006), whereas loss of GLUT3 is lethal as early as ED6.5 (Ganguly et al., 2007). This finding corroborates the fact that in human placentas GLUT3 is predominantly responsible for glucose uptake at the beginning of pregnancy. Decreased GLUT3 expression in heterozygous GLUT3-null embryos causes IUGR (Ganguly et al., 2007), but heterozygosity of GLUT1 seems not to affect fetal outcome (Ohtsuki et al., 2006). Thus, the mouse model clarified the hypothesis that GLUT3 is the candidate for complementing GLUT1 in placental glucose uptake. Moreover, rodent models have shown that maternal food restriction decreases placental levels of GLUT3 protein at term but does not affect the levels of either GLUT1 or GLUT4 protein. Therefore, GLUT3 may guarantee the glucose supply to the fetus, even with low maternal plasma glucose concentrations (Lesage et al., 2002). Similarly, a reduction of food intake in mice leads to a reduction in GLUT3 levels and a decrease in glucose transport function without altering GLUT1 expression (Ganguly et al., 2012).

A study using the vessel ligation model showed that the expression of GLUT1 transporters and Cx26 channels is downregulated in rat placentas at term (Nüsken et al., 2016), a finding that confirms the results of Das et al. (1998), who used a similar ligation model and found a downregulation in placental GLUT1 protein expression and reduced fetal glucose uptake. Therefore, regulation of GLUT1 seems to be downregulated if both nutrition and oxygen levels are strongly reduced. Interestingly, using a mouse model with strongly reduced food intake, Coan et al. (2010) found a reduction in GLUT1 expression at mid-gestation but an increase near term. Higgins et al. (2016) described a more intricate effect of hypoxia on glucose transport depending on the severity and the window of the hypoxic insult during pregnancy. Although 13% oxygen leads to an adaptation of the placental exchange area and an increase in the transfer of glucose and amino acids, maternal exposure to 10% oxygen during late gestation produces the opposite effect, with reductions in amino acid levels and glucose transport. Cuffe et al. (2014) found a reduction in the expression of GLUT1, insulin-like growth factor 2 (IGF2), and IGF-1 receptor (IGF1R) in placentas from pregnant mice kept under conditions of 17% hypoxia during midgestation to late term, but only in female fetuses. These animal studies point to a highly sensitive regulation of glucose transport in response to reductions in oxygen or food supply and to the time of application during pregnancy, with an adverse effect on the placenta in a sex-specific manner.

The results of studies of placental expression of GLUT transporters during human pregnancy affected by IUGR have also been conflicting. Several studies exhibited no evidence for an alteration in placental GLUT1 expression in IUGR pregnancies (Jansson et al., 2002). In contrast, Kainulainen et al. (1997) revealed that GLUT3 and GLUT4 are detected only at very low levels in human placentas and the total amount is even reduced in IUGR. Recently Janzen et al. (2013) found that GLUT3 protein expression is higher in the trophoblast of full-term IUGR placentas than in that of normal placentas but that there are no differences in GLUT1 or GLUT4 expression whereas GLUT1 and GLUT3 expression is increased when human trophoblast cells, primary cells, or cell lines are maintained under hypoxic conditions; this increase is probably mediated by hypoxia-inducible factor 1α (HIF-1α) (Esterman et al., 1997; Hayashi et al., 2004; Baumann et al., 2007). However, GLUT1 expression in the syncytiotrophoblast of placentas of women living at high altitude is significantly reduced (Zamudio et al., 2006). The differences in these results could be caused by the use of whole organs rather than cell lines.

Because of these conflicting results, the way in which GLUT1 and GLUT3 contribute to IUGR is still under debate, and the sensing of GLUT transporters seems to be dependent on the kind and severity of the maternal insult in human and rodents.

To achieve more insight into the effect of an insult on the regulation of glucose transporters, we must integrate the glucose metabolism of the placenta itself and the role of placental glycogen stores into this interplay for glucose release and transport to the fetus. Glycogen storage locations are found in both human and rodent placentas (Akison et al., 2017). In human placentas, glycogen is stored predominantly in the distal extravillous trophoblast at the placental-maternal interface (Shafrir and Barash, 1991) and in endothelial cells of fetoplacental vessels (Desoye et al., 2002). In mice and rats the glycogen cells are located as nests within the spongiotrophoblast layer. Mouse models have shown that stored glycogen is important for the high fetal demand for glucose in mid-gestation and at term (Coan et al., 2006; Oh-McGinnis et al., 2011). In human IUGR pregnancies, placental glycogen accumulation may reflect an inability to liberate glucose from the glycogen stores when needed, as a consequence of reduced numbers of glucose transporters to the fetus; these factors in combination contribute to fetal growth restriction. Thus, glucose and glycogen stores act as compensating partners that could explain controversial studies focusing only on glucose transfer and activity or those using cell lines in their studies. Another explanation for the differing results in glucose transporter regulation under conditions of fetal hypoxia leading to IUGR is based on the observation that the placenta exhibits high glucose consumption under conditions of hypoxia, and the enhanced glucose metabolism could also impede transplacental transport to the fetus without altering GLUT transporters (Challis et al., 2000; Zamudio et al., 2010). Furthermore, it has to be taken into account that the overall exchange area of the syncytiotrophoblast could be reduced by a smaller placenta or the uptake of glucose could be reduced by a decreased maternal blood flow without changing the expression of glucose transporters.

The GLUT isoforms not only exhibit differences in cell specificity but also are regulated by different stimuli, such as hypoxia, insulin, glucose availability, and IGF-1, as shown by *in vivo* or *in vitro* studies. As described above, IGF-1 regulates the expression of placental glucose and amino acid transporters; therefore, it is positively associated with fetal and placental growth. Baumann et al. (2014) showed that IGF-1 acts via microvillous or basal membrane receptors; this action increases the basal membrane expression of GLUT1, resulting in an increase in transepithelial glucose transport. Evidence of such a mechanism has recently been provided by Jones et al. (2013), who found that IUGR can be overcome with treatment by delivering adenoviral-mediated IGF-1 into the placenta; this treatment enhances the expression of several glucose transporters.

Glucose transport across the placenta seems to manage the mammalian target of rapamycin (mTOR) signaling pathway in human placentas. The mTOR signaling pathway is known as a placental sensor to regulate cell growth in response to the environmental supply of nutrients. Studies using cultured primary human trophoblast cells have shown that mTOR complex is regulated by glucose (Roos et al., 2009b). In addition, Brett et al. (2015) found an alteration of genes involved in the transport of fatty acid, amino acid, and glucose, as well as in mTOR signaling in pregnant women according to variations in maternal physical activity and diet composition. They observed a strong positive correlation between total sugar uptake and GLUT1 expression and an inverse correlation between total sugar uptake and both mTOR expression and IGF-1 expression, and it is well-known that IGF-1 signaling is inhibited in IUGR (Laviola et al., 2005; Street et al., 2011). To date there are only few studies about mTOR involvement in glucose transport regulation in rodent placentas. Placental GLUT1 and GLUT3 expression is downregulated by treating pregnant rats with synthetic glucocorticoids (Hahn et al., 1999), and excessive glucocorticoid concentrations caused by stress or exogenous administration in pregnant rats resulted in IUGR probably by altering placental glucose transport and upregulation of the mTOR signaling pathway (Vaughan et al., 2015).

### Amino acid transport across the placental barrier

An active transport mechanism across the placental barrier is necessary because amino acid concentrations are higher in the fetus than in the maternal compartment (Regnault et al., 2005). Many types of placental amino acid transporters are involved in the transport across rodent and human placentas (Lager and Powell, 2012), including neutral, cationic, and anionic amino acid transporters, which act partly sodium-dependent or sodium-independent (Regnault et al., 2005; Grillo et al., 2008; Desforges and Sibley, 2010).

The system A transporters consist of three sodium-coupled neutral amino acid transporter (SNAT) proteins, SNAT1 (SLC38A1), SNAT2 (SLC38A2), and SNAT4 (SLC38A4) (Zhang et al., 2015) and are found highly expressed on the apical with a weaker expression on the basal plasma membrane of the syncytial layer, all responsible for the transplacental amino acid transports. In rodents and humans results suggested that SNAT1 is most likely the major contributor to system A-mediated neutral amino acids uptake and that the remaining amino acid uptake is mainly mediated by SNAT4 in humans and by SNAT2 in rats (Takahashi et al., 2017) (Figures 1A,B). The release of SNAT mediated amino acid uptake to the fetal compartment at the basal side is probably mediated by the system L transporters (Jansson, 2001).

The transport of anionic amino acid is mediated by excitatory anionic amino acid transporters (EAAT1-4) (Matthews et al., 1998) and transport of cationic amino acid is mediated by the sodium-independent cationic amino acid transporter family (CAT) with CAT1 (SLC7A1), CAT2 (SLC7A2), CAT4 (SLC7A4), all found in human trophoblast cell lines (Kamath et al., 1999; Ayuk et al., 2000). Other Na(+)-independent amino acid transporters are represented by the LAT-1 (Light chain-type amino acid transporter 1) system which contributes to the placental amino acids exchange. System L is comprised of two transporters, each containing the long chain molecule 4F2hc and one of the short chain molecules, LAT1 or LAT2. LAT1 is predominately found on the apical plasma membrane and LAT2 on the basal membrane (Gaccioli et al., 2015). Localization of LAT2 on the basal membrane allows for the exchange between non-essential amino acids in the fetal circulation and essential amino acids in the human syncytiotrophoblast cytoplasm (Kudo and Boyd, 2001; Figure 1A). Because of the more complex structure of the rodent placental barrier with two syncytial layers, a direct comparison of their localization with the human placenta barrier is not yet possible. Although the gap junction channels are permeable for peptides up to 18 kD (Cieniewicz and Woodruff, 2010) nothing is known if the amino acids can cross the two syncytial layers of the trophoblast against the concentration gradient (Figure 1B). It is well-known that downregulation of placental amino acid transporters directly contributes to IUGR because active transport of essential amino acids in rodents and humans is necessary (Kusinski et al., 2010).

In rodents, placental system A transporter activity is well-known to be inversely correlated with placental weight; even reduced placental weight, which is not correlated with IUGR, required a higher level of transporter activity to compensate (Coan et al., 2008). Also reduced expression of EAAC1 and CAT1 accounts for the decrease in fetal, and placental weight upon maternal protein malnutrition in rats (Malandro et al., 1996).

Interestingly, Nüsken et al. (2016), using a bilateral uterine artery and vein ligation rat model that limits both oxygen and nutrient delivery to the fetus, found a downregulation of the neutral amino acid transporters SNAT1 and SNAT2, combined with growth-restricted pups exhibiting a phenotype similar to that of the low-protein model. However, this study also found that sham-operated rats exhibited reduced amino acid transporter expression and reductions in the body weight of the pups. These findings indicate that two separate insults, stress and reduced blood flow, both discharge into a common mechanism leading to IUGR.

Jansson and Powell (2006) showed that the downregulation of amino acid transport system A precedes IUGR in rats fed a low-protein diet and that the altered transporter system is linked to reduced levels of insulin, IGF-1, and leptin. Thus, nutritional deprivation in turn affects growth hormones, particularly those of the IGF-1 system (Bauer et al., 1998; Woodall et al., 1998), and this effect could have direct consequences for IUGR. These findings are corroborated by work of Constância et al. (2002) who generated the placental-specific *Igf2* knockout mouse (P0) by deletion of a paternally imprinted *Igf2* promoter; this mouse bears IUGR fetuses at term and led to a diminution in system A transport but an increase in Glut3 expression. Interestingly, placentas of P0 fetuses exhibited a reduction in placental size before the onset of IUGR. The late onset of IUGR is due to an adaptive increase in system A amino acid transporter activity at mid-gestation; this activity does not occur closer to term (Constância et al., 2002).

As in rodents, placental amino acid transport is decreased in humans with IUGR (Hayward et al., 2016). Researchers have found downregulation of specific amino acid transport proteins, such as system A in the placentas of SGA babies from the microvillous membrane of the syncytiotrophoblast (Mahendran et al., 1993). Studies have also shown that not all amino acid transporter systems act similarly in mice and humans. Kusinski et al. (2010), for example, showed that system B, a taurine transporter across the syncytiotrophoblast, exhibited less activity in mouse membrane vesicles than in the human microvillous membrane of the syncytiotrophoblast in growth restriction.

Like for glucose transport, the mTOR pathway and its downstream effector p70S6kinase1 (p70S6k1) in the human syncytiotrophoblast form an important regulatory link between growth-factor concentrations and the amino acid transport system especially the activity of system-L essential amino acid transporters (Roos et al., 2007, 2009a,b,c). Moreover, mTOR regulates system L amino acid transporters but not system A or taurine transporters; it also alters the placental leucine transporter responsible for IUGR (Roos et al., 2007). In contrast, Chen et al. (2015) recently reported that mTOR signaling regulates system A amino acid transport by modulating ubiquitination and plasma membrane trafficking, because inhibition of placental mTORC1 and mTORC2 signaling pathways decreases the expression of SNAT-2 protein in the syncytiotrophoblast microvillous plasma membrane of human IUGR placentas. Additional evidence that the mTOR pathway is also important in the rodent placenta came from studies by Rosario et al. (2011), who found that mTOR signaling is reduced in placentas of rats fed a low-protein diet together with decreased maternal levels of insulin, IGF-1, and leptin, as well as the phosphorylated signal transducer and activator of transcription 3 (STAT3) signaling, which is supposed to downregulate the expression and activity of all placental amino acid transporters. All of these findings point out that placental mTOR signaling functions as a nutrient-sensing pathway in rodents and humans and that impairment of this signaling pathway reduces the expression of amino acid transporters, with the consequence of IUGR.

### Fatty acid transport across the placental barrier

Altered fatty acid transport and lipid availability may add one more variable to the signaling field leading to IUGR and could also contribute to alterations in tissue development that result in chronic diseases later in adulthood (Daniel et al., 2016). It is known that maternal lipid profiles are important determinants of fetal growth (Kulkarni et al., 2013). Both saturated and unsaturated fatty acids, which are taken up by the placenta from the maternal circulation by diffusion, are fundamental precursors of various bioactive molecules.

Diffusion of lipids across the placenta along a concentration gradient seems to be insufficient to support the embryo, especially at the end of pregnancy. Thus, several proteins are associated with fatty acid transport: the fatty acid transport proteins (FATPs) comprise six, members, five of which have been found in the human rat placenta (Schaiff et al., 2005), the fatty acid translocase FAT/CD36, the fatty acid binding proteins (FABPs), and the fatty acid binding protein of the plasma membrane (FABPpm). FATP and FAT/CD36 are located in the apical and the basal membranes of the human syncytiotrophoblast and are thus responsible for lipid transfer to the fetus (Cunningham and McDermott, 2009; Lager et al., 2016) (Figure 1A). Kazantzis and Stahl (2012) reported that the cellular membrane FATPs, with the help of FAT/CD36, are important for the cellular uptake of long-chain fatty acids. In the rat cytoplasmic heart fatty acid binding protein (hFABP), FABPpm, FAT/CD36, and FATP are expressed in labyrinth zones of the rat placenta. The predominant expression of hFABP and FAT in the labyrinth zone of the chorioallantoic placenta implicates that hFABP and FAT/CD36 are involved in the transplacental transport of fatty acids from maternal to fetal compartments (Knipp et al., 2000). Nothing is known, however, about the precise localization of fatty acid transport system in the two syncytial layers of the rodent placental labyrinth and how lipids can pass the inter-syncytial space. Gap junction channels cannot serve this function because the pore is hydrophilic and water filled (Figure 1B).

A study using cultured primary term human trophoblast cells found that, upon a hypoxic environment, FATP2 expression increases, whereas FATP4 expression decreases (Biron-Shental et al., 2007). It seems important to note that the expression and activity of the proteins involved in fatty acid transport are influenced by insulin, IGF-1, and leptin, forming a network of interaction (Mousiolis et al., 2012).

Unlike the many studies of the human placenta, only a few studies have examined alterations in lipid transporter expression in rodent models of IUGR. Nüsken et al. (2016) used the arterial and vein ligation rat model and found that the expression of fatty acid transporters (hFABP, and FAT/CD36) is doubled in placentas of ligated and sham-operated rats but that the expression of FAT/CD36 protein is significantly increased only in ligated animals. However, unlike the amino acid transporter, lipid transporter expression was unchanged in sham-operated animals, a finding indicating that not all maternal insults, stress and reductions in nutrition and oxygen levels, produce similar alterations in transporter expression or activity (Nüsken et al., 2016).

Although the expression patterns of the various transporter molecules are similar, there are some differences between rodent and human fatty acid transport regulation, as indicated by the findings of Mishima et al. (2011). Hypoxia upregulates the expression of FATP2 and FATP4 in human trophoblast cells, whereas in the mouse FATP2 and FATP4 seem not to be essential for intrauterine fetal development, because embryos and placentas of both types of mice exhibit normal weight (Mishima et al., 2011).

Lipid uptake by the different proteins is modulated by insulin, IGF, and leptin network (Magnusson-Olsson et al., 2006; Mousiolis et al., 2012), and, in turn, regulates placental protein transporters as well as the mTOR signaling pathway (Roos et al., 2009a,b,c). Many reports have shown that the mTOR pathway is a positive regulator of trophoblast system A and system L amino acid transporter activities but only few studies describe the integration of mTOR signaling pathway in the lipid transfer. Oleic acid has been reported to activate mTOR signaling (Lager et al., 2014).

Saturated acids such as palmitine acid (PA) and unsaturated fatty acids such as docosahexaenoic acid (DHA) and oleic acid (OA) differentially affect trophoblast amino acid transport and cellular signaling. DHA counteract the OA stimulatory effect on amino acid transport and mTOR phosphorylation, whereas PA had no effect on the amino acid transport and the mTOR pathway (Lager et al., 2014).

Taken together, the findings of these studies indicate that mTOR signaling mediates nutrient sensing in the trophoblast directly or indirectly and serves as a key regulator of placental nutrient transporters, as postulated by Jansson et al. (2012).

Moreover, the fetal demand for cholesterol is high. Cholesterol is necessary for fetal growth later in gestation and is essential for cell membrane integrity and synthesis of steroid hormones as well as for activation of various signaling pathways such as the sonic hedgehog for appropriate brain development in early pregnancy (Chiang et al., 1996). However, knowledge about the consequences of disturbances in transport and metabolism for placental function and IUGR is still elusive. Though the precise route of transcellular transport is unknown, a number of the transporters—not all mentioned here—are involved (Burke et al., 2009). The placenta transports cholesterol from the maternal circulation to the fetus through cholesterol-carrying lipoproteins, primarily low-density lipoproteins (LDL), high-density lipoproteins (HDL), and very low-density lipoproteins (VLDL). Both LDL- and HDL-cholesterol have been shown to be taken up by the trophoblast of rodents and humans via its receptors and is secreted out of the basal side via protein-mediated processes (Burke et al., 2009; Woollett, 2011). Cholesterol transfer to plasma lipoproteins and efflux from the trophoblast to the fetal circulation is generally believed to rely on adenosine triphosphate–binding cassette (ABC) transporters. ABCA1 is highly expressed in the human placenta in various compartments, including the villous syncytiotrophoblast with ABCA1 on the microvillous and ABCG1 on the basal plasma membrane. Apical ABCA1 expression indicates a role in cholesterol efflux to the mother, while basal ABCG1 indicates the transport to the fetus (Aye et al., 2010). ABCA1 protein expression is significantly elevated in human syncytiotrophoblast of IUGR placentas but less in preeclampsia placentas (Körner et al., 2012; Baumann et al., 2013). This suggest that the regulation mechanisms of cholesterol transfer to the fetus is not only dependent on hypoxia.

Determining the consequences of decreased placental ABCA1 expression for placental function requires additional investigation (Körner et al., 2012). ABC transporter expression is induced by the liver X receptor (LXR) bound to the retinoid X receptor (RXR) upon oxysterol stimuli. Using human trophoblast cell lines and placental explants, Plösch et al. (2006, 2010) demonstrated that LXRA increased together with ABCA1 upon hypoxia, a finding that contradicts the findings of Körner et al. (2012). The generation of ABCA1-deficient mice, has revealed the high importance of the ABC1 transporter for mouse placental development. ABCA1 deficiency causes severe placental malformation resulting in retardation of embryo growth and loss of fetuses (Christiansen-Weber et al., 2000) and may serve as a manifold genetic tool for examining the role of ABCA1 in maternal-fetal cholesterol transport.

Placentas of rats fed a low-protein diet exhibit upregulation of numerous genes related to cholesterol and lipoprotein transport, as well as LXR/RXR and Farnesoid X receptor (FXR)/RXR (Daniel et al., 2016). This finding corroborates earlier findings showing that hypoxia causes enhanced FATP expression in humans (Mishima et al., 2011) and of FABP in rats (Nüsken et al., 2016); it also supports the hypothesis that these factors are crucial for organ development in mice, rats, and humans.

## Conclusions and future perspectives

This review has outlined similarities and differences in placental transport mechanisms in rodents vs. humans. Despite structural differences in the placentas of rodents vs. humans, most of the data are overlapping such as the expression of the most important transporters in the placental barrier (summarized in Figures 1A,B) and their functional mechanisms. The data further underline that rodent models play an important role in understanding placental nutrient transfer in the human under normal and pathological situations.

Despite all of the investigations about molecular pathways, such as altered expression and function of nutrient receptors, transporters, or hormones combined with dysregulated signaling cascades as causes of IUGR, the possibility of interventions for preventing IUGR is in its infancy. One reason for this lack of interventions is the absence of a clearly defined connection between a specific insult and a definite type of altered placental function that results in IUGR. On the other hand, we cannot exclude the possibility that these insults result in similar changes in placental signaling pathways and functions. For future interventions it would be very important to elucidate and discriminate these two options. At least some indications from animal experiments provide the fact that a wide variety of nutritional and environmental insults during pregnancy leads to IUGR; such insults include food restriction (Woodall et al., 1996), protein restriction (McMullen and Langley-Evans, 2005; McMullen et al., 2012), and high-fat feeding (Khan et al., 2005). These factors produce the same phenotypic outcome in the offspring: cardiovascular impairment (primarily hypertension) and impairment of nephrogenesis in adult life (Langley-Evans et al., 1999; Benz and Amann, 2010). Before medicating pregnant women to compensate for changes in the nutrient and oxygen transport of the placenta, it would be necessary to find reliable biomarkers indicating the types of placental alterations to develop defined targets for interventions including several dietary supplements. Moreover, detailed information about integrated pathways is available in humans and rodents (Ilekis et al., 2016) and the targets may be able to stimulate signaling pathways compensating for the adverse environment. One of the most important signaling cascade to compensate for an adverse environment is the growth factor signaling pathways of mTOR. mTOR appears to be a central manipulator, acting like a spider in a web in regulating multiple nutritional and metabolic signals in both humans and rodent models. Research findings support the conclusion that mTORC1 acts as a placental nutrient sensor and as a switch point that can be manipulated by various drugs. Although we must remember that some placental physiology and metabolism are in part differently regulated by rodents and humans, it appears that the main regulation strategy of the nutrient transport and signaling pathway of the placenta is comparable; however, comparison of the various studies is hampered by controversial results in both species. If we are to receive robust data, guidelines for IUGR experiments in animal models are necessary and are ultimately mandatory for developing and surveying new therapies for a crucial intervention during pregnancy in humans.

## Author contributions

EW has designed the main scientific content of the review and written the main parts. AG has written the part of glucose and fatty acid transport.

### Conflict of interest statement

The authors declare that the research was conducted in the absence of any commercial or financial relationships that could be construed as a potential conflict of interest.
